# Unveiling the Perspective on *Weissella confusa* as a Promising Biocontrol Agent Against Fusaria

**DOI:** 10.3390/microorganisms13030666

**Published:** 2025-03-15

**Authors:** Sukumaran Vipin Krishnan, Prakasan A. Anaswara, Kesavan Madhavan Nampoothiri, Szilvia Kovács, Cintia Adácsi, Ida Miklós, Szabina Király, István Pócsi, Tünde Pusztahelyi

**Affiliations:** 1 Microbial Processes and Technology Division (MPTD), CSIR-National Institute for Interdisciplinary Science and Technology (NIIST), Thiruvananthapuram 695019, India; vipinkrishnan05@gmail.com (S.V.K.); anaswaraprakash001@gmail.com (P.A.A.); 2 Academy of Scientific and Innovative Research (AcSIR), Ghaziabad 201002, India; 3 Food and Environmental Toxicology Research Group, Central Laboratory of Agricultural and Food Products, Faculty of Agricultural and Food Sciences and Environmental Management, University of Debrecen, H-4032 Debrecen, Hungary; kovacs.szilvia@agr.unideb.hu (S.K.); adacsi.cintia@agr.unideb.hu (C.A.); 4 Department of Applied Genetics, Institute of Biotechnology, Faculty of Science and Technology, University of Debrecen, H-4032 Debrecen, Hungary; miklos.ida@science.unideb.hu; 5 Department of Molecular Biotechnology and Microbiology, Institute of Biotechnology, Faculty of Science and Technology, University of Debrecen, H-4032 Debrecen, Hungary; kiraly.szabina@science.unideb.hu (S.K.); pocsi.istvan@science.unideb.hu (I.P.)

**Keywords:** lactic acid bacteria, *Fusarium*, deoxynivalenol, fumonisins, zearalenone

## Abstract

The biotechnological potential of the lactic acid bacterial genus *Weissella* has not been fully unearthed. Since *Weissella* have not been tested against Fusaria and their mycotoxins, newly isolated *Weissella confusa* strains were characterized and tested for their antifungal capacities on *Fusarium* plant pathogens. *W. confusa* BF2 and ML2 successfully inhibited *Fusarium verticillioides* NCIM 1100, *F. verticillioides* NCIM 1099, *Fusarium graminearum* MTCC 2089, and *Fusarium oxysporum* MTCC 284 in co-cultures. Ethyl acetate extracts of the cell-free culture supernatants (CFCS) of *W. confusa* also exhibited antifungal activity against the tested fungal cultures. The main mycotoxins of Fusaria were tested against the *Weissella* isolates. In MRS broth, *W. confusa* BF2 was resistant to the *Fusarium* mycotoxins (deoxynivalenol, zearalenone, T2, and fumonisin B1), while the ML2 strain showed 22.1–24.5% growth inhibition. Meanwhile, neither bacterium showed potential in mycotoxin reduction. The study highlighted that *W. confusa* BF2 and ML2 and their CFCS are suitable for *Fusarium* growth inhibition, as shown on surface-sterilized peanuts and wheat grains, but not for mycotoxin elimination.

## 1. Introduction

Phylogenetically, the *Weissella* genus belongs to the Firmicutes, class Bacilli, order *Lactobacillales*, and family *Leuconostocaceae* [1]. *Weissella confusa* [1] synonyms are *Lactobacillus confusus* [2] and *Lactobacillus coprophilus* subsp. *confusus* [3]. Bacteria ascribed to the genus *Weissella* are Gram-positive, catalase-negative, non-endospore-forming cells with rod-shaped or coccoid morphology [1,4] and have their place in the group of bacteria usually known as lactic acid bacteria (LAB). They are obligately heterofermentative with either D (−)- or a mixture of D (−)- and L (+)- lactic acid and acetic acid as the primary end products from sugar metabolism. Approximately 22 recognized species are within the genus *Weissella* [4,5,6].

The *Weissella* genus is increasingly recognized for its potential applications in diverse fields, from food to health care. However, the biotechnological potential of the genus *Weissella* has not been fully unearthed. Remarkably, *Weissella cibaria* and *Weissella confusa* are regarded as high producers of exo-polysaccharides exhibiting texturizing properties [5,6]. Some *Weissella* strains resisted low pH and bile salts and were isolated from healthy human feces, indicating their potential as probiotics. These strains also serve an essential role in food fermentation and preservation with various health benefits due to the production of several compounds like bacteriocins, hydrogen peroxide, and organic acids [5,6,7,8,9,10]. *Weissella* spp. are highly valuable in food preservation due to the production of bacteriocins, which exhibit antimicrobial properties against food spoilage bacteria. *W. cibaria* strains possess antagonistic activity against pathogens like *Streptococcus pyogenes*, *Staphylococcus aureus*, *Streptococcus pneumoniae* [7] and *Listeria monocytogenes* [8]. *W. confusa* strains could fight *Helicobacter pylori* [9] and multidrug-resistant *Escherichia coli* [10]. Several bacteriocins have been defined in *Weissella hellenica* and *W. confusa* strains (reviewed by [11]) and may be utilized as bio-preservatives. Some *Weissella* strains can decarboxylate polymeric phenolic compounds, resulting in improved bioavailability. The use of *Weissella* genus or *W. confusa* as an industrial starter is unknown because, despite *Weissella* spp. being prospective good starters for food fermentation, they are not recognized as GRAS (Generally Recognized As Safe) organisms (FDA, US) or given Qualified Presumption of Safety (QPS) status (EFSA, EU). Their antibiotic resistance patterns and the possibility of biogenic amine production or infection risks partially clarify this disregard [5,12,13]. However, *Weissella* spp. can be considered safe according to international standards due to the lack of virulence genes and toxic metabolite production and may achieve GRAS status in the future [14,15].

A rising challenge for green-labeled food pushes us to avoid applying chemicals in agricultural production systems; thus, identifying biocontrol agents is essential for the sector. Recent research suggested that LAB are the best choice as biocontrols for mitigating fungal contamination and mycotoxins [16,17,18,19,20,21,22,23]. More research is needed to identify this Gram-positive group’s potential for agricultural utilization.

Plant-pathogenic and mycotoxin-producing Fusaria are economically important filamentous fungi that contaminate various crops. Among diverse *Fusarium* mycotoxins, deoxynivalenol (DON), fumonisins (FUM), zearalenone (ZEA), and T2/HT2 toxins are reported to cause risks [24,25]. While *Fusarium verticillioides* is a known pathogen, it can also exist within plants as an endophyte, typically without inducing any disease symptoms. In conducive environments, maize is susceptible to seedling blight, stalk, or ear rot when infected with *F. verticillioides*. Beyond just lowering crop yields, it also generates FUM mycotoxins, posing a risk to humans and other organisms [26,27]. *Fusarium graminearum* is one of the major fungal pathogens responsible for Fusarium head blight. *F. graminearum* synthesize DON during infection, a virulence factor that promotes Fusarium head blight spread [28]. *Fusarium oxysporum* is a fungus living in soil that generates several toxins, including fumonisins and fusaric acid, while it produces other less significant mycotoxins like beauvericin, enniatins and moniliformin [1]. It does not create trichothecenes or ZEA [29] and can attack numerous crops [30].

LAB produce acids and other antifungal agents, inhibiting *Fusarium* growth in low pH environments [31]. LAB can also positively affect plant cultures; for example, *W. cibaria* DA2 cell-free culture supernatant (CFCS) positively impacted sweet corn quality and resistance [32]. *Weissella paramesenteroides* demonstrated intense antifungal activity against *Aspergillus flavus* and reduced aflatoxin B1 production [33], while *Weissella soli* strains exhibited low aflatoxin B1 binding potential [34]. It was concluded that galactan exopolysaccharides could increase the aflatoxin B1 binding potential of the genus, such as *W. confusa* KR780676 [6].

We aimed to isolate LAB from different sources and investigate LAB and *Fusarium* interactions for *Fusarium* growth control. Since the literature on the interaction of *Fusarium* and *Fusarium* mycotoxins with the *Weissella* genus was rare, in this study we intended to present our results on *W. confusa* isolates’ potential as antifungal LAB for *Fusarium* disease control and our studies on possible *Fusarium* mycotoxin reduction.

## 2. Materials and Methods

### 2.1. Microorganisms

Lactic acid bacteria (8 isolates) were isolated on MRS medium plates at 37 °C from neonatal feces and bovine milk samples collected from Trivandrum, Kerala, India. Pure bacterial cultures were obtained by preparing decimal dilutions, plating them in MRS medium, incubating at 37 °C for 24–48 h and re-streaking isolated colonies. *Fusarium oxysporum* MTCC 284, *Fusarium verticillioides* NCIM 1100 (ATCC 14164), *Fusarium verticillioides* NCIM 1099 (ATCC 12616), and *Fusarium graminearum* MTCC 2089 were used for the antagonism studies. To grow the fungi on a solid medium, PDB (Hi-Media, Mumbai, Maharashtra, India) grown cultures were streaked on PDA plates (Hi-Media, Mumbai, Maharashtra, India) and incubated at 30 °C for 5–7 days.

### 2.2. Characterization of the Bacteria

Gram-staining and catalase tests were performed as basic identification. Gram-positive and catalase-negative cultures were then assessed for the zone of inhibition formed on modified MRS-calcium carbonate (0.8% *w*/*v* CaCO_3_) plates (Hi-Media, Mumbai, Maharashtra, India) and incubated at 37 °C for 48 h [35,36]. Based on their inhibition zone, selected colonies were streaked onto MRS agar to produce pure cultures (Hi-Media, Mumbai, Maharashtra, India).

For DNA isolation, strains were grown in MRS broth at 37 °C for 48 h and harvested by centrifugation (13,600× *g* for 5 min). Genomic DNA was extracted using a bacterial genomic DNA extraction kit (G-Spin Intron Biotechnology, Seongnam, Republic of Korea) according to the manufacturer’s instructions. The absorbance ratio A260/A280 determined the DNA concentration and purity. The PCR amplification of the 16S rRNA bacterial barcode sequence was conducted using 16S rRNA universal bacterial primers [1492R: 5′-GGT TAC CTT GTT ACG ACT T-3′, 515F: 5′-GTG CCA GCM GCC GCG GTA A-3′] (IDT, Leuven, Germany) [36] at 55 °C annealing temperature. PCR reaction with Phusion Hot Start II High–Fidelity DNA Polymerase (Thermo Fisher Scientific Life Technologies Inc., Carlsbad, CA, USA), cleanup and sequencing were performed according to Krishnan et al. [36]. The sequences were analyzed using MEGA 11 (version 11.0.13.) software. A homology-based identification was performed with BLASTn: https://blast.ncbi.nlm.nih.gov/Blast.cgi (accessed on 22 March 2024). The sequences were submitted to the NCBI GenBank [37]. Using Advanced mode with the default parameters, a phylogenetic tree was built with PCR product sequences at Phylogeny.fr (http://www.phylogeny.fr, accessed on 23 January 2025). The sequences of *Latilactobacillus sakei* and *Weissella confusa* were downloaded from the NCBI database https://blast.ncbi.nlm.nih.gov/Blast.cgi (accessed on 23 January 2025).

### 2.3. Antagonism Test

A solitary bacterial colony was taken from a fresh MRS plate and mixed with 250 µL of sterile distilled water. The resulting suspension was then spread across half of a fresh MRS plate using a sterile cotton swab, and the plate was incubated at 37 °C for 48 h. The fungi were grown in PDA. To assess antifungal activity, a fungal disc was placed on the other half of the MRS plate, previously inoculated with bacteria, using a sterile cork borer. After incubation at 30 °C for 5–6 days, the plates were compared to control plates to observe any inhibition of fungal growth by the bacteria. Fungal inhibition was calculated as the percentage reduction (I%) in colony diameter compared to untreated controls. All standard deviations were less than 5%.

### 2.4. Preparation of Bioactive Extracts and Well Diffusion Assay

A 250 mL MRS broth was inoculated with 16–24 h-old bacterial culture (OD_600nm_ = 1) and incubated at 37 °C for 48 h at static conditions. After incubation, the supernatant was collected by centrifugation at 8590× *g* for 15 min. The supernatant was mixed with ethyl acetate (EA) (in ratio 1:2) in a 2 L conical flask and shaken for an hour at room temperature. The organic layer was collected and concentrated using a rotary evaporator (Heidolph Instruments GmbH, Schwabach, Germany). The semi-viscous concentrated sample was collected and adequately suspended. The concentrated crude extract was tested for antifungal activity against *Fusarium* spp. using well-diffusion assays.

For the tests, *Fusarium* spp. were grown in PDB and incubated for 2–3 days at 30 °C [36]. To conduct the well-diffusion test, the fungal suspensions prepared in PBS were evenly spread on MHA plates and 100 µL of the crude extract was added per well (n = 3). After incubation at 30 °C for 72 h, the mean of the inhibition zone and standard deviations were determined (n = 3).

### 2.5. Mycotoxin Detection with HPLC Method

HPLC measurements were performed on Thermo Scientific Dionex Ultimate 3000 (Dionex Softron Ltd., Germering, Germany) HPLC equipment. For all measurements, Biopure mycotoxin standard solutions (Romer Labs, Tulln, Austria) were used [38]. Mycotoxin sample preparation and measurement in the reverse phase were conducted according to Krishnan et al. [36]. Thermo Scientific Dionex Chromeleon 7.2 Chromatography Data System (CDS) software (Dionex Softron Ltd., Germering, Germany) was used for data collection and evaluation. The LOD was 0.01 mg/kg for DON and 0.001 µg/kg for ZEA. A linear range of up to 50 mg/kg was detected. The relative standard deviation was calculated as the absolute value of the coefficient of variation (RSD), and in all cases, it was found to be below 10% [36].

### 2.6. Mycotoxin Detection with ELISA Method

Mycotoxin detection in competitive ELISA tests was performed for FB1 and T-2 mycotoxins. For the AgraQuant^®^ Fumonisin ELISA kit, the LOD was 0.2 mg/kg, LOQ was 0.025 mg/kg, and the quantitation range was 0.2–5 mg/kg (Romer Labs, Tulln, Austria). For the AgraQuant^®^ T-2 Toxin ELISA test, the LOD was 0.01 mg/kg, the LOQ was 0.020 mg/kg, and the quantitation range was 0.02–0.5 mg/kg (Romer Labs, Tulln, Austria). The samples were measured at 450 nm (n = 3, RSD < 5%) using a BioTek Synergy HTX Multimode Reader (BioTek, Winooski, VT, USA) [36].

### 2.7. Mycotoxin Resistance Tests

The resistance tests were performed according to Krishnan et al. [36]. The isolates were inoculated into MRS broth (Scharlab, Barcelona, Spain) and incubated at 37 °C for 16 h. A microtiter plate made with 200 μL MRS medium was inoculated to obtain a low-density culture (OD_630nm_: 0.1–0.2). BIOPURE mycotoxins (Romer Labs, Tulln, Austria) were added to the cultures to a 2 mg/L final concentration. The bacteria were incubated with mycotoxins at 37 °C for 24 h in a microtiter plate reader (BioTek Synergy HTX Multimode Reader, BioTek, Winooski, VT, USA). The optical density was taken hourly at 630 nm after intense shaking (30 sec). Data (n = 4) were analyzed in Gen5 version 3.05 software (BioTek) and Microsoft Excel. A 5% growth inhibition was considered the minimal level statistically different from the control (*p* < 0.05).

### 2.8. Mycotoxin Reduction Tests

Mycotoxins (BIOPURE, Romer Labs, Tulln, Austria) were diluted with MRS broth. All mycotoxin-supplemented 16 h MRS LAB cultures (37 °C) were incubated for 1 h at 25 °C by shaking (250 rpm), then centrifuged (13,600× *g* for 5 min, 4 °C). The supernatants were removed, treated with methanol in a 1:1 ratio, vortexed at high speed, filtered with a 0.45 μm pore Spartan syringe filter (Whatman GmbH, Dassel, Germany), and analyzed with HPLC (2.5) [36].

### 2.9. Biocontrol Efficacy of CFCS on Grains

MRS broth was inoculated with 16–24 h old bacterial culture (OD _600nm_ = 1) and incubated at 37 °C for 48 h at static conditions. Peanuts and wheat were washed with distilled water, surface disinfected with 1 *w*/*v* % sodium hypochlorite for 1 min, washed with sterile distilled water, and air dried. A mix of 10 g surface-sterilized peanut, and 2 mL of the CFCS (10× concentrated) of *W. confusa* were incubated at room temperature for 3 h. Then the peanuts were contaminated with 200 µL of *Fusarium* conidiospore suspension and incubated at 30 °C for 7 days [39]. The control sample, designed to assess the effect of the bacterial treatment, consisted of 10 g of peanuts treated with 2 mL of sterile MRS broth. Disease incidence (DI) was calculated from the number of infected peanuts (1).

DI = (number of infected peanuts × 100)/total number of peanuts treated(1)

Samples of 40 g of surface-sterilized wheat grains and 5 mL of CFCS (10× concentrated) were incubated at room temperature for 3 h. Then, 1 mL of a fungal suspension was inoculated into each dish and mixed to ensure homogenization. To create the control samples, the substrate was inoculated with 5 mL of sterile MRS broth. The Petri plates were incubated at 30 °C for 7 days and grains were visually checked for fungal growth [40]. Disease incidence was determined based on fungal mycelial coverage in the Petri dish.

### 2.10. Statistical Analyses

Growth data analysis was performed in Gen5 3.05 software (BioTec, Winooski, VT, USA) and significance analysis was done in Microsoft Excel version 2501 Analysis ToolPac Add-in, where Tukey’s test (*p* ≤ 0.05) was performed.

## 3. Results

### 3.1. Characterization of Bacterial Isolates

**Figure 1:**
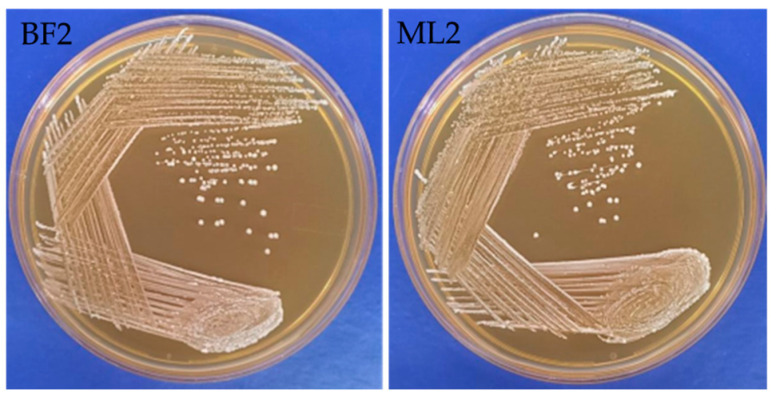
Macromorphological characterization of *Weissella confusa* BF2 and ML2 strains.

From neonatal feces (three isolates) and bovine milk samples (five isolates), we isolated eight LAB. From these isolates, based on their antagonistic activity against the Fusaria strains [*Fusarium oxysporum* MTCC 284, *Fusarium verticillioides* NCIM 1100 (ATCC 14164) and *Fusarium verticillioides* NCIM 1099 (ATCC 12616), *Fusarium graminearum* MTCC 2089], BF2 and ML2 isolates were selected for further antifungal studies. The isolates were tested using biochemical characterization and Gram staining. ML2 and BF2 isolates were Gram-positive, non-motile, short rod-shaped bacteria with flat, circular, creamy-white colonies (Figure 1).

**Figure 2:**
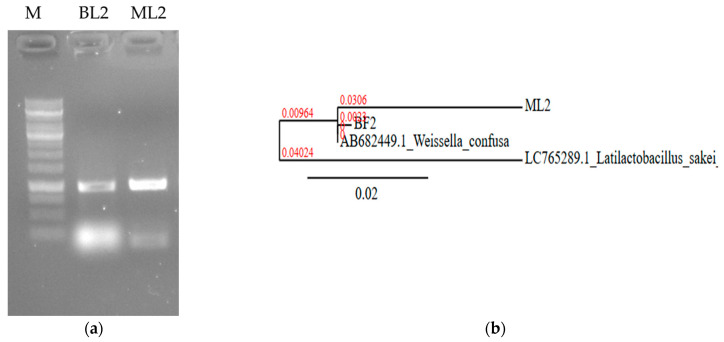
Nucleic acid-based identification of BL2 and ML2 isolates. (**a**) Sequence amplification with 16S rRNA specific primers. M: 1 kb DNA marker. (**b**) Neighbor-joining phylogenetic tree based on 16S rDNA sequence showing the relationship with *Weissella confusa* NBRC 106469 gene:AB682449.1. *Latilactobacillus sakei* LC765289.1 was used as an outgroup. The scale bar indicates the evolutionary distance, and the number along the tree branch indicates the bootstrap value.

They were non-spore-forming and catalase-negative (Table 1). The isolates were identified based on 16S rRNA nucleic acid PCR (Figure 2a). Sequences were uploaded to the NCBI database under accession number PP535080.2 as *W. confusa* strain ML2 16S ribosomal RNA gene, partial sequence (688 bp DNA) and PP029339.2 as *W. confusa* strain BF2 16S ribosomal RNA gene, partial sequence (560 bp DNA). The sequence of ML2 showed 99.47% homology (score: 1018) with 99% coverage with *W. confusa* HA1 16S RNA partial sequence (accession number: MK255068.1). The BF2 sequence was found to have 100% homology and 100% coverage with several *W. confusa* strains; e.g., *W. confusa* 1632 16S rRNA, partial sequence (score: 1035; accession number: MT597518.1). Phylogenetic analysis revealed that the BF2 and the ML2 sequences are closely related (Figure 2b).

### 3.2. Fusarium Antagonism

**Figure 3:**
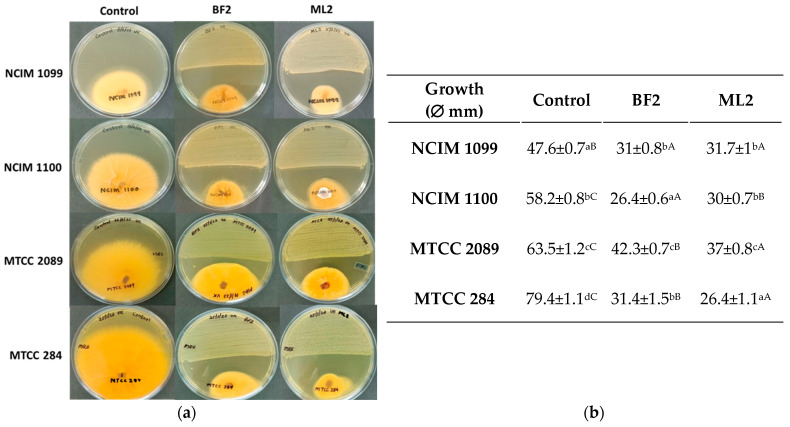
*Weissella confusa* isolates show antagonism against *Fusarium* cultures. (**a**) The activity of *Weissella confusa* strains (BF2 and ML2) against *Fusarium verticillioides* NCIM 1099, *F. verticillioides* NCIM 1100, *F. graminearum* MTCC 2089, and *F. oxysporum* MTCC 284. (**b**) The mean inhibition-zone diameter and standard deviation are shown (n = 3). Significant differences are shown with different letters (*p* < 0.05). Lowercase letters compare fungal growth results gained under the same treatment. In contrast, uppercase letters show significant differences between treatments of the same fungal cultures.

The antagonism test showed significantly different inhibition by *W. confusa* BF2 and ML2 strains against *Fusarium* cultures on MRS agar (Figure 3). Against *F. verticillioides* NCIM 1099, 34% inhibition was shown by both isolates without significant differences. However, *F. verticillioides* NCIM 1100 was more sensitive as 55% and 46% inhibitions were calculated for BF2 and ML2 strains, respectively. Against *F. graminearum* MTCC 2089, 34% and 42% inhibitions were found for ML2 and BF2 strains, respectively. *F. oxysporum* MTCC 284 was the most sensitive to the *Weissella* strains, as 63% and 69% inhibitions were detected for the two strains.

**Figure 4:**
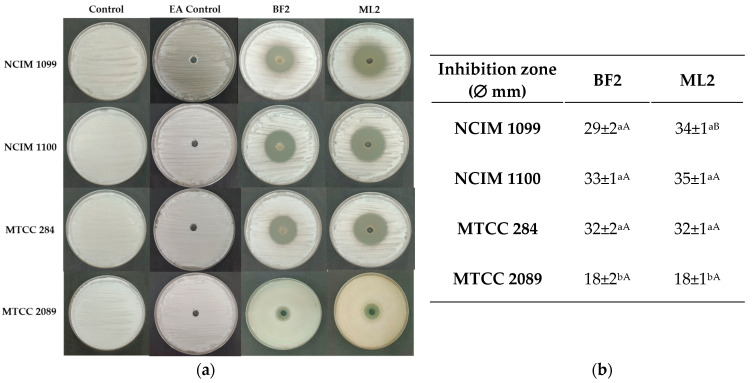
Diffusion test. (**a**) Antifungal activity of ethyl acetate extracts of CFCS of *Weissella confusa* BF2 and ML2 against *Fusarium verticillioides* NCIM 1099 and NCIM 1100, *F. oxysporum* MTCC 284, and *F. graminearum* MTCC 2089 strains. EA: ethyl acetate. (**b**) The average diameters of the inhibition zones, along with their standard deviations, are presented (n = 3). Statistically significant differences (*p* < 0.05) are indicated by different letters. Lowercase letters denote differences in inhibition zones produced by varying concentrations of the same strain’s cell-free culture supernatant (CFCS) extract. Uppercase letters signify differences in inhibition zones produced by different strain extracts on the same fungal strain.

A well-diffusion test with EA extract of the CFCS exhibited the highest antifungal activity against *F. verticillioides* strains (NCIM 1099 and NCIM 1100), and more against *F. oxysporum* MTCC 284 than against *F. graminearum* MTCC 2089 (Figure 4). Between the two strains, no significant difference was detected in antifungal effect against the fungal cultures except against NCIM 1099, while the CFCS EA extract of *W. confusa* isolate could inhibit both *F. verticilloides* strains and *F. oxysporum* without significant differences. *F. graminearum* MTCC 2089 showed higher resistance against the CFCS extract’s antifungal effect.

### 3.3. Mycotoxin Resistance and Reduction

The growth inhibition of *Weissella confusa* strains by different *Fusarium* mycotoxins and the elimination of the same mycotoxins were also tested. A significant growth reduction (22.1–24.5%) was detected for *W. confusa* ML2 with all tested mycotoxins (DON, ZEA, T2, FB1) at 2 mg/L toxin concentration, and there were no statistically significant differences within the treatments (*p* > 0.05). Interestingly, *W. confusa* BF2 showed resistance to all tested mycotoxins. Mycotoxin reduction was also characterized in liquid cultures, and the same 2 mg/L mycotoxin concentration was tested, but no mycotoxin reduction could be observed.

### 3.4. Biocontrol Tests

**Figure 5:**
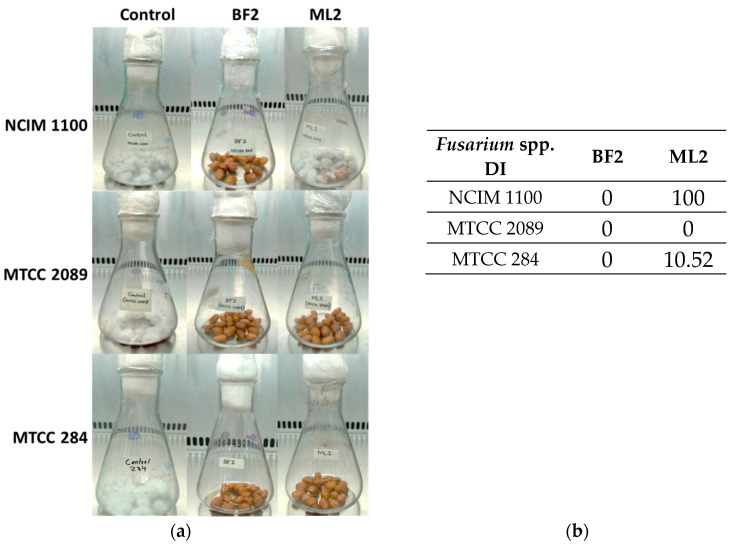
The antifungal effect was tested on surface-disinfected peanuts. *Fusarium verticillioides* NCIM 1100, *F. oxysporum* MTCC 284, and *F. graminearum* MTCC 2089 strains were tested against cell-free culture supernatants (CFCSs) of *Weissella confusa* BF2 and ML2 strains. (**a**) Pictures illustrating Fusaria growth on the surface of peanuts that had been disinfected, taken after a 7–day incubation. (**b**) Evaluation of *Fusarium* spp. growth with CFCSs. Disease incidence (DI) was calculated for the peanuts.

Biocontrol efficacy experiments with peanuts revealed that *W. confusa* ML2 has limited antifungal activity against *F. verticillioides* NCIM 1100 (Figure 5a), while applying BF2 in peanut treatment was successful against all three *Fusarium* species. The disease incidence (DI), which for the controls was 100%, was zero for all of the BF2 isolate treatments, while for the ML2 strain, it was 100% with NCIM 1100, 10.52% with MTCC 284, and zero with MTCC 2089 (Figure 5b).

**Figure 6:**
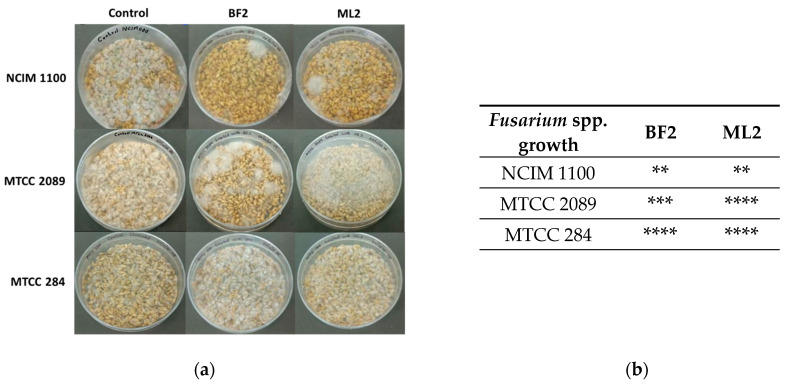
The antifungal effect was tested on surface-disinfected wheat grains. *Fusarium verticillioides* NCIM 1100, *F. oxysporum* MTCC 284, and *F. graminearum* MTCC 2089 strains were tested against the CFCSs of *Weissella confusa* BF2 and ML2 strains. (**a**) After seven days of growth, the presence of contaminating Fusaria was examined. (**b**) The extent of fungal contamination was then evaluated: **: colony formation by Fusaria (or 25%); ***: colony formation by Fusaria (or 50%); ****: complete contamination by Fusaria compared to controls.

Surface-disinfected wheat grains were also treated with bacterial isolates and infected with *Fusarium* sp. (Figure 6). It was concluded that the resulting antifungal effect was variable. Contrary to the above findings on peanuts, *F. verticillioides* NCIM 1100 was sensitive to *W. confusa* ML2 and BF2. *F. graminearum* MTCC 2089 was more sensitive to *W. confusa* BF2, while *F. oxysporum* MTCC 284 was not sensitive to the CFCS treatments.

## 4. Discussion

*Weissella confusa* is not a well-known industrially applied LAB. It was recently identified from sorghum, wheat semolina, sourdough, etc., [5], and now from fecal matrix and milk. It effectively inhibited *Penicillium* spp., *Aspergillus nidulans*, *Rhodotorula* spp., and *Endomyces fibuligera* [41,42].

LAB strains can combat mycotoxigenic filamentous fungi (e.g., *Fusarium* spp., *Penicillium* spp., *Aspergillus* spp.) by competing for resources and releasing a range of anti-microbial compounds, specifically small molecules and bacteriocin-like substances [43,44]. Of course, the production of *W. confusa*’s antifungal compounds depends on environmental conditions such as growth media, temperature and incubation time, pH, and nutritional factors [45,46]. Here, similar experimental settings were applied to compare the strains’ performances.

*W. confusa* strains were recently found to inhibit *Fusarium culmorum* spore proliferation [35]. Furthermore, ethyl acetate (EA) extracts of *Lactiplantibacillus plantarum* CFCS inhibited *Fusarium* metabolism [47]. *W. confusa* ML2 showed a similar antagonistic effect to the BF2 strain against all tested Fusaria. The well-diffusion assay using EA extracts of CFCS of *W. confusa* strains (ML2 and BF2) showed intense antifungal activity against *Fusarium* strains. Based on the literature, antifungal activity was connected to polylactic acid (PLA), 2-hydroxy-4-methylpentanoic acid, and other organic acid synergism [48] and also to some bacteriocins [11,49]; *Weissella cibaria* PS2 and certain *Lactobacillus* species were found to produce antifungal carboxylic acids, including benzoic, vanillic, azelaic, hydrocinnamic, and hydroxybenzoic acids, which were isolated from their culture media [21]. Initial research suggests that certain *Weissella* strains possess antifungal properties, demonstrating varied levels of effectiveness against fungal diseases like *Verticillium* wilt and *Mucor folium* [50]. These studies indicate *Weissella*’s ability to hinder the growth of filamentous fungi, highlighting its potential as a growth-inhibiting agent. The cell-free supernatant produced by *Weissella cibaria* BYL4.2 hindered the growth of *Penicillium chrysogenum* by preventing the germination of its conidiospores. This suggests that the antifungal properties of the CFCS are due to the presence of bioactive compounds within it [51]. However, in our case, further experiments are needed to identify effector molecules against *Fusarium* spp. in these *Weissella* strains.

The composition and structure of the plant material were also essential factors in the modelled biocontrol. Peanuts have a different chemical composition than wheat grains, affecting antifungal compound diffusion, bacterial performance, and, presumably, fungal spore attachment to the surface. This could explain the difference in the antifungal effects in peanuts and wheat against the same *Fusarium* species by the same strain.

The present study investigated *W. confusa* isolates and concluded that the isolates had potential as antifungal LAB for *Fusarium* mold control. Also, it was determined for the first time that these *Weissella* isolates were unsuitable for *Fusarium* mycotoxin reduction. Therefore, their application could only be suggested at the beginning of mycelial growth before mycotoxin production. It also concluded that the application of the bacteria is matrix-dependent, and other extraction methods also need to be tested before all antifungal factors can be successfully analyzed.

## Figures and Tables

**Table 1:** Identification and characterization of lactic acid bacteria isolates.

LAB Strain		Source	Gram Staining	Catalase Assay	MRS+ CaCO_3_	Accession Number *
BF2	*Weissella confusa*	Newborn feces	+	-	+	PP029339
ML2	*Weissella confusa*	Cow milk	+	-	+	PP535080

* Accession numbers in NCBI databank for 16S ribosomal RNA gene partial sequences.

## Data Availability

The original contributions presented in this study are included in the article. Further inquiries can be directed to the corresponding authors.

## Abbreviations

The following abbreviations are used in this manuscript:

CFCS	Cell-Free Culture Supernatant
DON	Deoxynivalenol
ZEA	Zearalenone
FB1	Fumonisin B1
FDA	U.S. Food and Drug Administration
EFSA	European Food Safety Authority
LAB	Lactic Acid Bacterium
PDA	Potato Dextrose Agar
PDB	Potato Dextrose Broth
MRS	de-Man-Rogosa-Sharpe
MHA	Mueller- Hinton Agar
NCIM	National Collection of Industrial Microorganisms
ATCC	American Type Culture Collection
MTCC	Microbial Type Culture Collection and Gene Bank
EA	ethyl acetate
